# Pharmacogenomics and health disparities, are we helping?

**DOI:** 10.3389/fgene.2023.1099541

**Published:** 2023-01-23

**Authors:** Sherin Shaaban, Yuan Ji

**Affiliations:** ^1^ Department of Pathology, University of Utah School of Medicine, Salt Lake City, Utah, United States; ^2^ ARUP Laboratories, Salt Lake City, Utah, United States

**Keywords:** genetics, pharmacogenomics (PGx), disparities, equity, precision medicine

## Abstract

Pharmacogenomics has been at the forefront of precision medicine during the last few decades. Precision medicine carries the potential of improving health outcomes at both the individual as well as population levels. To harness the benefits of its initiatives, careful dissection of existing health disparities as they relate to precision medicine is of paramount importance. Attempting to address the existing disparities at the early stages of design and implementation of these efforts is the only guarantee of a successful just outcome. In this review, we glance at a few determinants of existing health disparities as they intersect with pharmacogenomics research and implementation. In our opinion, highlighting these disparities is imperative for the purpose of researching meaningful solutions. Failing to identify, and hence address, these disparities in the context of the current and future precision medicine initiatives would leave an already strained health system, even more inundated with inequality.

## Introduction

The COVID-19 pandemic has helped expose the staggering rates of health disparities both domestically and world-wide, yet those disparities have long been demonstrated. The root causes continue to be debated with the goal of identifying future applicable solutions (Braveman and Gottlieb, 2014; Weinstein, 2017; Lee et al., 2020; Landrigan et al., 2021). Precision medicine initiatives carry a lot of promise in addressing these disparities with the potential to reduce morbidity and mortality for millions of people, while decreasing the cost and improving the quality of health care (Ginsburg and Phillips, 2018; Sisodiya, 2021). Without concerted efforts towards inclusion of minorities and disadvantaged populations in the research and development of such initiatives, what would have been potentially promising, could end up being a new roadblock that widens the already existing gaps. One of the most promising areas of precision medicine is pharmacogenomics (PGx), the science that utilizes genetic variation to individualize drug therapy. While the field has been growing exponentially over the last few decades with great potential, there is no evidence that the pattern of the implementation or utilization of PGx is predictive of a path guaranteeing health equity in such efforts.

Health disparities are preventable differences in the burden of disease, injury or opportunities to achieve optimal health that are experienced by socially disadvantaged populations (CDC. Health Disparities, 2022). In the United States (US), federal regulations define socially disadvantaged individuals as those who have been subjected to racial or ethnic prejudice or cultural bias within the American society because of their identities as members of groups and without regard to their individual qualities. These prejudices could be due to sex, age, location, occupation, race, ethnicity, religion, citizenship status, disability, and sexual orientation or gender identity (CFR, 2022; Ivers, 2022). The grave toll of health disparities far exceeds the direct harm to the disadvantaged individual into the whole society in terms of lost productivity, increased health care costs, and excess morbidity and mortality (LaVeist et al., 2011; Bush, 2018; Essien et al., 2021). Addressing these health disparities is not merely an issue of aspiring to achieve equity, but an investment into reducing avoidable health-care costs and towards building productive healthy societies. The US congress-commissioned report published in 2022, addressing the representation in clinical trials and research states that: “Despite greater diversity, deep disparities in health are persistent, pervasive, and costly. As the United States becomes more diverse every day, without major advancements in the inclusion of underrepresented and excluded populations in health research, failing to reach these growing communities will only prove more costly over time” (Bibbins-Domingo and Helman, 2022). Here, we review some of the determinants of health disparities in the context of PGx research and implementation.

### PGx and implications of genetic ancestry

According to the National Human Genome Research Institute, genetic ancestry is defined as the information about the people that an individual is biologically descended from, including their genetic relationship (https://www.genome.gov/genetics-glossary/Genetic-Ancestry). People of different genetic ancestries have different frequencies and severity of disorders as well as variance in response to therapeutic agents (Ortega and Meyers, 2014; Ramamoorthy et al., 2015; Gu et al., 2017; Shah and Gaedigk, 2018). PGx can help identify the genetic variation underlying interindividual differences in response to medication and therefore can better instruct which medication and which dosage to prescribe. Yet, most available research studies and clinical trials in the field of PGx have been conducted in either individuals of European descent or relied on genome-wide association studies (GWAS) which have long been criticized for lack of diversity (Haga, 2010; Sirugo et al., 2019; Magavern et al., 2022; Davis and Limdi, 2021). In a review article investigating the diversity in precision medicine and PGx research studies, the authors analyzed 146 studies, of which 104 were conducted in north America (71%), 26 in Asia (18%) and 16 studies were conducted elsewhere (Africa, Australia, Europe, and South America) (Popejoy, 2019). Given that over 77% of the world population reside in Asia and Africa (UN, 2022), the concentration of such studies outside these continents carries significant implications on their clinical utility outside North America, or even for non-European populations residing within North America (Popejoy and Fullerton, 2016; Popejoy, 2019; Sirugo et al., 2019). Taking Warfarin as an example, the drug was approved as an anticoagulant in the year 1954 (Lim, 2017), yet proper dosing remains a main challenge given its extremely narrow therapeutic index (Johnson and Cavallari, 2015). In 2007 the US Food and Drug Administration (FDA) relabeled warfarin with dosing recommendations based on genetic variation in *CYP2C9* or *VKORC1* for optimization (Bodin et al., 2005; FDA Coumadin, 2022). In 2016 the Clinical Pharmacogenetics Implementation Consortium (CPIC) updated their PGx-guided Warfarin dosing to add *CYP4F2* and rs127777823 to *CYP2C9* and *VKORC1*. Given that CPIC relies on available literature, the limited diversity among participants in the studies used to generate the guidelines, is an acknowledged limitation of such effort (Johnson et al., 2017). To put this in context, in the United States, the second largest racial group after white Caucasians is the Hispanics/Latinos which made up 18.9% of the population, while the multiracial population was the fastest growing group based on the latest census (https://www.census.gov/) (Roman et al., 2020; Nicholas Jones et al., 2020). Individuals of a Hispanic origin, as well as African Americans (AA), are considered to be at an especially high risk for poor outcomes after anti-coagulation therapy with warfarin (Birman-Deych et al., 2006; Shen et al., 2007; Shen et al., 2008), yet they remain largely underrepresented in trials aiming at developing dosing algorithms for Warfarin (Bress et al., 2012; Duconge et al., 2015). Furthermore, the Hispanics are an admixed population of Europeans, Native American and West-Africans (Bryc et al., 2010; Baran et al., 2012) and therefore extrapolating the findings of PGx studies conducted among Europeans to such an admixed population carries the risk of undermining the validity of any evidence that supports the implementation of PGx and precision medicine and remains a flawed practice (Ramos et al., 2012; Claudio-Campos et al., 2015; Grinde et al., 2019; Lee et al., 2019). Similarly, findings from the few studies conducted among AAs should not be directly extrapolated to other black populations such as sub-Saharan Africans or Afro-Brazilians given that the genetic architecture of AAs is distinct from that of other Africans (Zakharia et al., 2009; Dandara et al., 2022). Moreover, the development of therapeutics relies on evidence from clinical trials conducted mainly in non-Hispanic Whites or less frequently among Asian populations. Therefore, African, Hispanic, or native American-specific variants are often missed during drug discovery and development and the significance of these variants will only be realized upon release of such drugs to individuals of the underrepresented populations with the development of adverse reactions (Flores et al., 2021; Venkatakrishnan and Benincosa, 2022). In addition to the need for prioritizing enrollment of large numbers of participants from less-studied populations for GWASs and clinical trials, alternative genome-wide approaches such as admixture mapping or utilization of ancestry informative pharmacogenetic loci to allow for incorporating data reflecting the genetic diversity of different ancestral backgrounds is equally critical (Enoch et al., 2006; Ramos et al., 2014; Yang et al., 2021). While gene-based dosing models have been developed, they are proven to be stronger when the data are corrected for admixture (Alzubiedi and Saleh, 2016; Shendre et al., 2018). A growing number of PGx studies focusing on biogeographically defined populations particularly among less studied groups such as Native Americans, Africans or South Asians populations are to be applauded (Ortega and Meyers, 2014; Bonifaz-Pena et al., 2014; Hariprakash et al., 2018; Nagar et al., 2019; Ahsan et al., 2020)

### Sex and gender-based PGx

Despite the robust evidence of varied response to medication between men and women; one cannot help but wonder why women continue to be subject to more adverse drug events, compared to men, with women having 1.5- to 1.7-fold greater risk of developing such adverse events (Rademaker, 2001; Soldin and Mattison, 2009; Zucker and Prendergast, 2020; FDA, 2021; Madla et al., 2021). This could very well result from treatment protocols relying on clinical trials dominated by male participants with disregard of the influence of sex and gender on drug safety and efficacy (Manteuffel et al., 2014; Ravindran et al., 2020). The difference in response to drugs between sexes has been attributed to many factors including biological differences in pharmacodynamics and pharmacokinetics (e.g., differences in absorption, distribution, metabolism, and excretion (ADME) genes, smaller volume of distribution, higher body fat in women, receptor numbers or binding), or to biological processes such as pregnancy and menopause (Soldin and Mattison, 2009; Mezzalira and Toffoli, 2021). While drug therapy is prevalent during pregnancy, how and why drug disposition is altered in pregnant women remains poorly studied and not all commercially available medications are tested for safety and efficacy during pregnancy (Buhimschi and Weiner, 2009; Jeong, 2010; Ito, 2016; Haas et al., 2018; Betcher and George, 2020). Taking another stage of female life, menopause is inevitable in all women, yet variations in timing, symptoms, and their severity, as well as needs and response to menopausal hormonal therapy varies significantly (Minkin, 2019). Estrogen remains the most effective medication used in menopause to treat its symptoms as well as to prevent serious related diseases such as cardiovascular diseases, osteoporosis, or even early death (Moyer et al., 2016; Paciuc, 2020). The influence of genetic variation on estrogen efficacy and metabolism has been well studied, yet genetically-based algorithms for estrogen administration and dosing relevant to its contribution to increased risk of development of venous thromboembolic events remain lacking (Wall et al., 2014; Moyer et al., 2018). Clinical trials to better understand the genetics underlying this thrombotic susceptibility are needed and could lead to establishing the safety of estrogen use among women who do not share that genetic risk (Herrington and Klein, 1985; Wall et al., 2014; Vinogradova et al., 2019; Abou-Ismail et al., 2020).

A new growing area of interest in PGx, is the study of the effect of variations in sex chromosomes between men and women (e.g., X-chromosome inactivation, gene mutations, differences in number of microRNAs or epigenetic deregulation, etc.) that could explain variations in drug response in general or response to certain classes of medications such as increasing resistance to cancer immunotherapy (Care et al., 2018; Irelli et al., 2020; Mezzalira and Toffoli, 2021).

In addition to inadequate studies addressing safety or proper dosing during various stages of a woman’s life, women’s under-representation in drug clinical trials remains a significant hurdle. The FDA and the National institute of health (NIH) published policies and guidelines to encourage women’s participation and inclusion in research and clinical trials (Nasem Women and Health Research, 1994; NIH, 2017; FDA Research, 2019). Despite these efforts having shown relative success in increasing recruitment among white women, this has not similarly translated to increase in recruitment of women of color (Camidge et al., 2021; Bierer et al., 2022). Larger scale PGx studies with adequate enrollment of female participants, including underserved women, as well as the emerging science of gender medicine or sex-based medicine could potentially be the answer to addressing the existing sex gap in the outcomes of treatments and/or toxicity (Mauvais-Jarvis et al., 2021; Mezzalira and Toffoli, 2021).

### Treatment access and socioeconomics inequalities

Individuals on the lower scale of socioeconomic status (SES) determinants e.g., income, education or racial ancestry are similarly subject to health disparities including less access to proper medication or diagnostic testing (Plumper et al., 2018; Ji et al., 2020; Trivedi et al., 2020; Salmond and Dorsen, 2022). This can be demonstrated for example by the case of direct oral anticoagulants (DOACs). While DOACs have become the standard of care for patients with deep vein thrombosis compared to Vitamin K antagonists, low-income and black patients consistently receive less prescriptions of DOACs even when they are insured (Nathan et al., 2019). Similarly, utilization of DOACs remain limited in African countries due to its unaffordability because of patent laws, in addition to lack of clinical trials to address its safety in African populations (Semakula et al., 2021; Dandara et al., 2022). With a growing body of studies of DOACs PGx, efforts to enroll minorities, which most probably would also fall on a lower level of SES, is of paramount importance. Additionally, if the landscape of reimbursement practices and insurance coverage for genetic testing including PGx does not improve, test access inequities will persist (Lee et al., 2018; Qureshi et al., 2022). Another example is cancer mortality rates and newer anti-cancer drugs. While the overall US mortality from all cancers declined by 26% from 1990 to 2015, the interventions to decrease that rate has not been uniform in terms of ancestry, region of residence or SES (Siegel et al., 2011; Robbins et al., 2012; Siegel et al., 2015; Ma et al., 2019). The utilization of human epidermal growth factor receptor 2 (HER2)-targeted therapies such as trastuzumab demonstrates the intricacies between SES and health disparities. (HER2)-targeted therapies have proven to be highly effective at treating breast cancer (Loibl and Gianni, 2017; Kay et al., 2021), yet clear racial and socio-economic disparities exist with regards to the receipt of such an effective medication, with black women being 25% less likely to receive trastuzumab than white women, with that percentage becoming even higher among poorer patients. The single most significant factor determining not receiving HER-2 therapies is lack of financial resources (Reeder-Hayes et al., 2016; Adusumilli et al., 2017). (HER2)-targeted therapies have many side effects including risk for cardiotoxicity. These side effects occur at particularly higher rates among AA women and in patients with additional risk factors such as Diabetes and Hypertension which in turn are prevalent in communities with lower SES including among poor white individuals (Gaskin et al., 2014; Glover et al., 2020; Price-Haywood et al., 2020; Al-Sadawi et al., 2021). Studies to decipher the genetic contributors to the heterogeneity in response and the side-effects of (HER2)-targeted therapies and other anti-neoplastic drugs are evolving including novel approaches utilizing PGx. Unfortunately, most of these studies continue to be conducted among majority white Caucasian populations (Wells et al., 2017; Garcia-Pavia et al., 2019; Jeibouei et al., 2019).

Unsurprisingly and given how intertwined race/ancestry is to socioeconomic resources, it was inevitable to repeatedly reference race and ancestry in this section that was meant to address the relation between SES and PGx access or utilization (Ribisl et al., 1998; NIH, 2004; Williams et al., 2016).

### Pediatric to geriatric PGx

Our knowledge about the clinical utility and cost-effectiveness of PGx among the pediatric population remains limited even for medications with available guidelines. CPIC has so far published 26 gene/drug clinical practice guidelines using evidence extracted from studies conducted mainly on adult individuals. About 50% of these drugs, either the whole class or individual drugs within these classes, have not been studied in children or have limited evidence of safety in pediatric populations. Extending these recommendations to the pediatric populations and adolescents remains controversial (Neyro et al., 2018; Roberts et al., 2021; CPIC, 2022). Fortunately, efforts to better understand the potential utility for PGx implementation among the pediatric population are ongoing (Namerow et al., 2020; Ramsey et al., 2021; Roberts et al., 2021). Various models have been adopted by different institutes, from single gene to panels tested either preemptively or reactively while offering point of care electronic clinical decision support (eCDS) to clinicians (Johnson et al., 2013; Haidar et al., 2019). Concurrently, consortia such as the Sanford Children’s Genomic Medicine Consortium consisting of ten children’s’ hospitals across the US, has integrating PGx into pediatric care as a major goal of the consortium’s efforts utilizing genomics in pediatric medicine (Gregornik et al., 2021).

Similarly, knowledge gaps still exist with regards to PGx and drug-gene associations in the elderly populations. Morbidity, mortality, and health care costs due to adverse drug reactions (ADRs) in the elderly is a major public health concern although many of which are predictable and avoidable (Onder et al., 2013; Bozina et al., 2020; Roman et al., 2020; Hoel et al., 2021). With a growing aging population, the economic burden of management and hospitalizations due to ADRs will only be exacerbated (Maher et al., 2014; Formica et al., 2018; Perez-Jover et al., 2018; Malki and Pearson, 2020). Evidence is growing to support the utility of PGx in guiding treatment regimens in older patients (Bozina et al., 2020; Inventor and Paun, 2021). Significantly higher rates of hospitalizations are observed in patients with polypharmacy (5 or more drugs) who harbor large number of PGx polymorphisms compared to those with polypharmacy and no or less genetic variants, while the rates of hospitalizations decrease and cost savings per patient increase if PGx-guided treatment is followed (Finkelstein et al., 2016a; Finkelstein et al., 2016b; Brixner et al., 2016). While polypharmacy is a common problem among the aging population, extrapolating the value of PGx-guided treatment to all polypharmacy patients regardless of age, would be a reasonable practice. PGx studies with the goal of establishing recommendations for genetic testing in older patients either pre-emptively or reactively upon hospitalization is an endeavor worth consideration.

### Healthcare settings: Primary care and rural communities

While most of drug prescriptions occur within primary care settings and despite robust evidence supporting the utility of PGx in medicine optimization, extending PGx implementation into primary care settings specially within rural communities remains challenging given the current models of clinical practice (Sudia, 2016; Dearing and Cox, 2018; Rollinson et al., 2020). Most PGx programs implemented to date are conducted within urban health care systems or large academic institutes (Houwink et al., 2015; Dawes, 2020; Leitch et al., 2022). Adding the current limited reimbursement available for PGx testing and the lack of providers comfort or literacy with PGx ordering to already existing problems in the rural communities such as physicians and health care providers shortages, lower socio-economic standards, less insurance coverage, and limited access to specialists, and then PGx implementation would easily be seen as an unattainable endeavor (Kogan et al., 2018; Johnston et al., 2019; Richman et al., 2019; Empey et al., 2021; Leitch et al., 2022). Yet the value of PGx to these resource-poor settings, in terms of improving the quality of healthcare by decreasing costs, reducing ADRs, and better management of polypharmacy should override such existing hurdles. To overcome PGx implementation barriers, innovative approaches need to be considered in such settings, such as improving access using telehealth tools or considering population-guided approaches to PGx (Patrinos, 2010; Mette et al., 2012; Naik et al., 2020). Additionally, given the novelty of PGx implementation in clinical practice, the availability of educational resources regarding PGx testing and related guidelines for both healthcare providers and patients becomes imperative (Haga, 2017; Amara et al., 2018). Without efforts to overcome the existing barriers to implementation of PGx and other personalized medicine initiatives in primary care and rural settings, one more layer of inequality in access to care will be added to an already underserved population.

## Discussion

With the current rates and patterns of population growth in the US, health disparities that currently cost up to $320 billion annually, could grow to $1 trillion by 2040 (Asif Dhar et al., 2022). This is obviously unsustainable. Despite scientific and technological advancements that improved health outcomes in the US, prevalence of diseases and death rates remain significantly higher among certain disadvantaged populations. Precision medicine aims at improving health through the concept of preventing and treating diseases relying on individuals’ or populations’ specific genetic or environmental make-up and shifting away from empirical management (Collins and Varmus, 2015). In the same context, pharmacogenomics (PGx), a main component of precision medicine, aims at improving the clinical outcomes of pharmacotherapy. In addition to the hope that precision medicine and PGx initiatives would improve health outcomes, it was projected that inadvertently it will also help address existing health gaps (Griffith, 2020). We chose some of the well-studied determinants of health such as genetic ancestry, sex, and socioeconomic status, as well as less commonly investigated ones, such as age and healthcare setting, to review if PGx implementation in the recent years have shown success or promise towards addressing health disparities. For all those determinants, and despite areas of success or active research, major challenges continue to exist. There was limited evidence of coordinated efforts or strategies designed to implement PGx and other precision medicines programs that take into account inclusion and implementation within underserved or disadvantaged populations. To be able to garner the benefits of PGx, structural changes are needed in the way we conduct PGx research, from the point of choosing participants of diverse backgrounds, to the setting where the studies are conducted to ensure accessibility, to how evidence-based guidance is developed ensuring inclusivity. Engaging with patient advocacy groups and with community partners such as local clinicians and religious leaders to set priorities and strategies for addressing local needs helps build trust with the targeted communities. Addressing individual barriers of recruitment by offering compensation, transportation options, flexibility in scheduling as well as multi-linguistic material and plain language consents and study information are needed. Increasing outreach through expanding telemedicine technologies and utilizing social media platforms to improve health literacy and education campaigns is another strategy to increase engagement. Additionally, diversifying the research team to include lay persons from the targeted community, and encouraging minority trainees and early career faculty to be part of or lead research efforts is postulated to increase trust and enhance enrollment. Funding agencies can also play a role by incentivizing minority populations enrollment such as through supplemental awards for innovative approaches to recruit individuals from rural or underserved communities. Moreover, assigning a score for inclusion of women or minorities could be another approach that would positively impact recruitment and retention. Retraining of study section reviewers and scientific officers to identify bias could also be needed. Lastly, Institutional Review Boards (IRBs) should ensure research studies have enrolled people who represent the groups affected by the condition or disease studied and require researchers clearly outline their recruitment strategies (Brooks et al., 2015; Clark et al., 2019; Strauss et al., 2021; Thakur et al., 2021).

Moreover, many barriers to the implementation of PGx into clinical practice need to be addressed. Integration into clinical workflows need to be improved, efficient, user-friendly clinical decision support tools need to be developed, terminology and practice need to be standardized, cost-effectiveness needs to be proven, and lastly, clinician education is a cornerstone for any successful adoption or implementation (Klein et al., 2017; Amara et al., 2018; Giri et al., 2019). These barriers are expected to be even harder to address in communities with less or limited resources. Additionally, without regulatory and legislative efforts towards improving reimbursement for PGx testing as part of the effort to improve overall access to genomic medicine, the high out-of-pocket costs would be an additional obstacle for implementation within underserved populations.

Most of the work discussed in this review originates from the United States or the global north. Needless to say, the challenges and limitations identified and discussed with regards to the implementation of PGx or other precision medicine programs in relation to health disparities will be present at even larger scales in the global south and developing countries that have much limited resources or access to new innovations (Chong et al., 2018; Shih et al., 2022). Without meaningful partnerships between the north and the south, and without creative solutions, these underprivileged regions of the world will be further deprived of tools that carry a lot of promise to improve efficiency and guarantee equity in healthcare.
